# Deep Learning Model for Intracranial Hemangiopericytoma and Meningioma Classification

**DOI:** 10.3389/fonc.2022.839567

**Published:** 2022-03-03

**Authors:** Ziyan Chen, Ningrong Ye, Nian Jiang, Qi Yang, Siyi Wanggou, Xuejun Li

**Affiliations:** ^1^ Department of Neurosurgery, Xiangya Hospital, Central South University, Changsha, China; ^2^ Hunan International Scientific and Technological Cooperation Base of Brain Tumor Research, Xiangya Hospital, Central South University, Changsha, China

**Keywords:** hemangiopericytoma, meningioma, magnetic resonance imaging, deep learning, classification

## Abstract

**Background:**

Intracranial hemangiopericytoma/solitary fibrous tumor (SFT/HPC) is a rare type of neoplasm containing malignancies of infiltration, peritumoral edema, bleeding, or bone destruction. However, SFT/HPC has similar radiological characteristics as meningioma, which had different clinical managements and outcomes. This study aims to discriminate SFT/HPC and meningioma *via* deep learning approaches based on routine preoperative MRI.

**Methods:**

We enrolled 236 patients with histopathological diagnosis of SFT/HPC (n = 144) and meningioma (n = 122) from 2010 to 2020 in Xiangya Hospital. Radiological features were extracted manually, and a radiological diagnostic model was applied for classification. And a deep learning pretrained model ResNet-50 was adapted to train T1-contrast images for predicting tumor class. Deep learning model attention mechanism was visualized by class activation maps.

**Results:**

Our study reports that SFT/HPC was found to have more invasion to venous sinus (*p* = 0.001), more cystic components (*p* < 0.001), and more heterogeneous enhancement patterns (*p* < 0.001). Deep learning model achieved a high classification accuracy of 0.889 with receiver-operating characteristic curve area under the curve (AUC) of 0.91 in the validation set. Feature maps showed distinct clustering of SFT/HPC and meningioma in the training and test cohorts, respectively. And the attention of the deep learning model mainly focused on the tumor bulks that represented the solid texture features of both tumors for discrimination.

## Introduction

Intracranial hemangiopericytoma (HPC) is a rare type of neoplasm developing from meningeal mesenchyme around vessels. Considering the overlapping molecular characteristics (1–3), the 2016 World Health Organization (WHO) classification of tumors of the central nervous system (CNS) combined HPC and solitary fibrous tumor (SFT) into one term SFT/HPC and assigned three grades within the entity (4). As such, this low proportion of intracranial tumors has a high risk of recurrence and systemic metastasis (5–7). Once diagnosed, SFT/HPC must be treated aggressively with a more detailed surgical treatment followed by radiotherapy and chemotherapy due to its malignancy of infiltration, peritumoral edema, bleeding, and bone destruction (8–11). However, it might be difficult to discriminate SFT/HPC from meningioma because of the different incidences but similar characteristics on clinical and radiological manifestations (**Supplementary Figure S1**) (12). On the contrary, not all meningiomas need to be treated aggressively. Therefore, precise distinction between SFT/HPCs and meningiomas are essential before surgery or therapy.

Previous studies have revealed that MRI-based imaging may contribute to the diagnosis of SFT/HPC (13–15). Radiologically, SFT/HPCs exhibit more aggressive behaviors like necrosis and bone erosions and heterogenous enhancement (16). And preoperative multimodal MRI images could supply sufficient information on tumor location, size, and peritumoral tissues for surgical planning. Previous quantitative analysis provides effective markers such as apparent diffusion coefficient (ADC) values in diffusion-weighted imaging (DWI) and the degree of intratumoral susceptibility signal intensity (ITSS) in susceptibility weight imaging (SWI) (14, 15, 17). However, SFT/HPC has a very low incidence, so that physicians might neglect the prescription of multimodal imaging. And these multimodal images are not always obtainable due to the machine-hour shortage and patients’ economic condition in many developing countries. Preoperative classification by routine MRI images is urgently needed.

Artificial intelligence approaches for routine MRI images have been proven to be efficient ways to achieve semantic segmentation of lesions and extraction of multidimensional information (18–20). State-of-the-art deep learning architectures such as convolutional neural network (CNN) have powerful performance in brain tumor classification, objection, and segmentation (21–23). And another advantage is to implement transfer learning that uses large pretrained model weights and fine-tunes the classification layers to obtain higher accuracy with few data.

In this study, we retrospectively collected data from patients from Xiangya Hospital with histopathologically confirmed SFT/HPCs and meningiomas. The aim was to adopt a pretrained deep learning neural network model ResNet-50 (24). By implementing the deep learning algorithms through single-modal conventional MRI images, our model achieved a high accuracy of preoperative diagnosis of SFT/HPCs and meningiomas. Hence, it can assist in surgical planning and treatment after the operation.

## Materials and Methods

### Clinical Cohort and Data Acquisition

**Figure 1:**
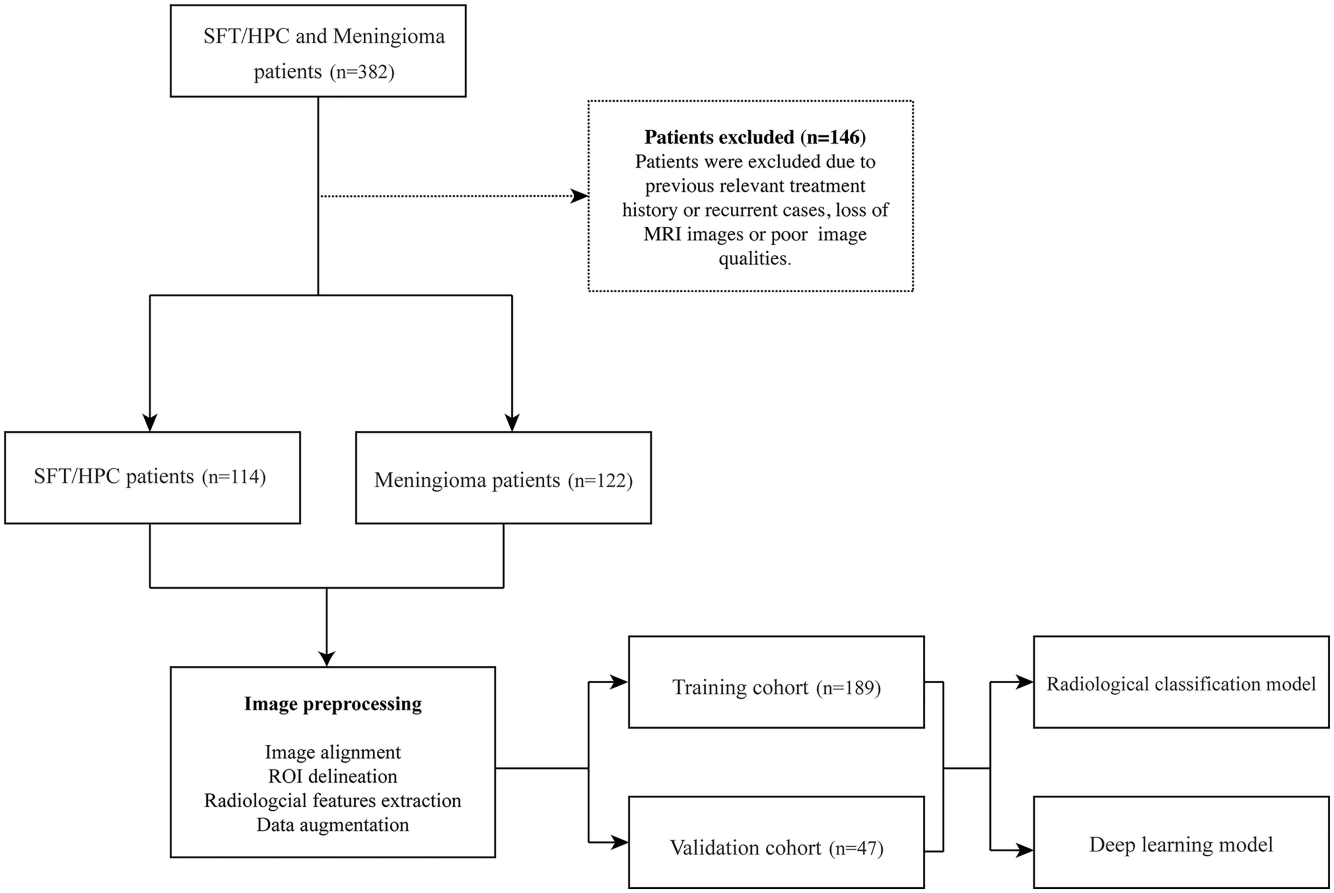
Flowchart of the whole study.

In our retrospectively study, a total of 236 patients with MRI data were enrolled in Xiangya Hospital from 2010 to 2020, with their clinical and pathological data collected from the Electronic Medical Record System. Considering that meningioma is way more common in our center, we selected similar numbers of patients to prevent model overfitting. Among them, 114 cases were pathologically diagnosed with SFT/HPC and 122 cases were pathologically diagnosed with meningioma. Exclusion criteria included previous relevant treatment history or recurring cases; patients without MRI images in our hospital or poor image qualities (**Figure 1**). Brain MRI was performed as part of routine clinical care on scanners from various manufacturers with different magnetic field strengths (**Table 1**) and acquisition parameters. This study was approved by the institutional review board of our hospital.

**Table 1:** Clinical scanners used in the study.

	SFT/HPC (n = 114)	Meningioma (n = 122)	*p*-value
**All manufacturers**			0.451
Total at 1.5T	81	68	
Total at 3T	33	36	
**3.0T Scanners**			0.847
SIEMENS	5	5	
GE Medical Systems	28	31	
**1.5T Scanners**			0.925
SIEMENS	53	58	
TOSHIBA	18	19	
Alltech Medical Systems	10	9	

HPC, hemangiopericytoma; SFT, solitary fibrous tumor.

### Imaging Preprocessing

For each patient, presegmentation image registration was performed with T1, T2, T1-weighted contrast-enhanced (T1C), and T2-weighted fluid-attenuated inversion recovery (FLAIR) images. Affine images were coregistered into the same geometric space using the Elastic toolbox (25). Voxels of different sets of images were resliced into an average size of 0.52 mm × 0.52 mm × 4.74 mm. All the sequences of images were used for the segmentation of tumors, peritumoral edemas, and cysts. ITK-SNAP, an open-source 3D image analysis software (26), was implemented for delineating tumor boundaries in a semiautomated fashion on a slice-by-slice basis. All regions of interest (ROIs) containing the main disease components were manually delineated on each MRI image by two neuroradiologists (NY and NJ) who had 5 and 10 years of combined experience in neurosurgery and brain tumor imaging, respectively. They were blinded to the patients’ medical information.

We evaluated the interobserver (reader 1 vs. reader 2) and intraobserver (reader 1 twice) reproducibility of lesion labeling by calculating the interclass and intraclass correlation coefficients (ICCs). For interobserver reproducibility, reader 1 and reader 2 segmented the lesions independently and they were blinded to each other’s segmentations. In addition, and for intraobserver reproducibility, reader 1 repeated the segmentation procedure within 1 week of the first analysis. Generally, ICC >0.80 indicated a good agreement for segmentation.

Simultaneously, we extracted the radiological factors including tumor boundary, bone erosion, dural sign, T1C enhancement patterns, venous sinus invasion, cystic components, and peritumoral edema. And we adapted a logistic regression to train a radiological diagnostic model for classification.

### Deep Learning Training and Validation

**Figure 2:**
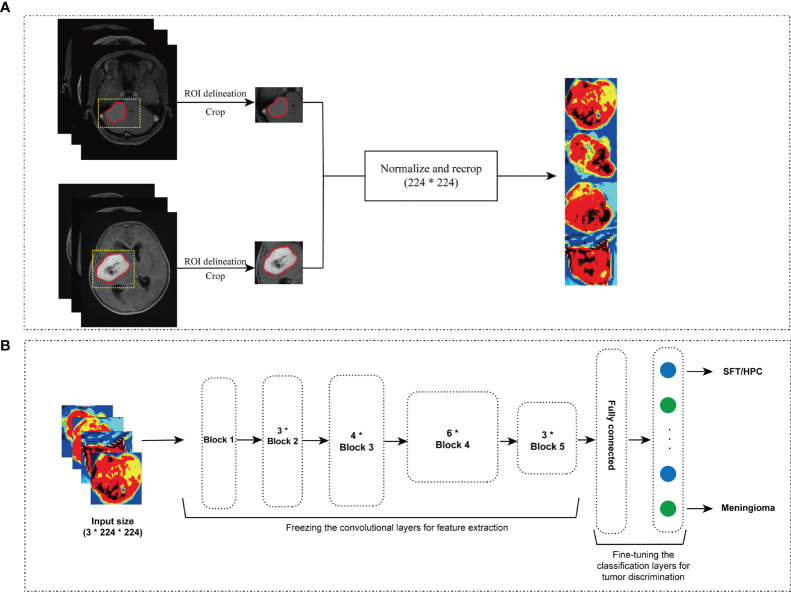
Image preprocessing and model training. Regions of interest (ROIs) were extracted from the aligned images, and the jet colormap was applied to grayscale MRI images, followed by the use of 5 image augmentation techniques **(A)**. After preprocessing, input image size was reshaped to 3 × 224 × 224. ResNet-50 convolutional layers were frozen, and the last classification layers were retrained for solitary fibrous tumor/hemangiopericytoma (SFT/HPC) and meningioma discrimination **(B)**.

ResNet-50 pretrained model was adapted to train the classification model, and we selected the center slice for each lesion to build our datasets. To fit the pretrained initial weights of 3 color channels, we applied the Jet colormap to convert the gray-level images into RGB images, then data augmentation was performed to prevent overfitting and extend the datasets. Concretely, 5 data augmentation approaches were used by *TorchIO* (27) including random flip random noise, random motion, random blur, and random ghosting. Finally, all images were normalized and recropped to 3 × 224 × 224 initial input size as expected by the model and divided into batches by batch size 16 for more efficient training (**Figure 2**). In this study, we compared different sequences including T1, T1C, and T2 and found superior model performance with a single T1C. Thus, we only used T1C and chose the center image of ROI in axial slice as input data. Given that T1C-based MRIs are commonplace among clinical protocols for patients with SFT/HPC or meningioma, our model would be broadly applicable.

We trained our model on an Ubuntu 18.04 computer with 1 Intel Core i9-7940 CPU using an NVIDIA GTX 1080Ti 11GB GPU, with 256 GB available system RAM. Training in all categories was run for 300 epochs by an SGD optimizer with momentum 0.9 and weight decay 4e-5 and cross entropy loss function. To fine-tune the pretrained model, we froze the convolutional layers and retrained the final fully connected classification layer. Learning rate was initially set as 2e-5 in the frozen layers and 1e-5 in the classification layer and utilized a decay rate of 0.9 for each of the 4 steps until the model reached convergence. In this study, we split our data into training set and validation set according to a 4:1 ratio (training cohort = 189, validation cohort = 47).

### Feature Analysis

We applied classification activation maps (CAMs) to visualize network attention. Internal mechanisms of deep learning algorithms have often been referred to as a “black box.” Implementation of CAMs could improve transparency and understand the operations and attentions of the model. We applied Smooth Grad-CAM++ (28) that uses the gradients of the target concepts to produce a coarse localization heatmap highlighting the important regions in images for predicting the concept for model visualization. Specifically, in any class *c*, Grad-CAM firstly computed the gradient of the score *y^c^* before softmax with respect to feature maps *A^k^*, then random samples in a neighborhood of inputs are taken to smooth the feature maps, and gradients flow back to obtain the importance weights from *A^k^*. To produce Smooth Grad-CAM++, we calculated the gradient of the ground truth with respect to the last layer before classification and used the *pytorch-grad-cam* github repository (https://github.com/jacobgil/pytorch-grad-cam). And we calculated the distance from the activation center to the center or edge of the tumor to compare the difference of the tumor recognition patterns.

We also extracted feature maps of the last layer before classification in the ResNet model and analyzed them by an unsupervised algorithm t-distributed stochastic neighbor embedding (t-SNE). This showed similarity between data points to joint probabilities and reduce the number of dimensions of image features depending on the non-linear function. And t-SNE was also applied to visualize high-dimensional radiological factors.

### Statistical Analysis

We used Student t and χ^2^ tests to evaluate differences in patient demographics between data split. Deep learning model performance was also assessed using positive predictive value (PPV), negative predictive value (NPV), sensitivity, specificity, f1-score, receiver-operating characteristic curve area under the curve (AUC), and average precision (AP) score. And *p* < 0.05 was considered statistically significant. All statistical analysis and visualization were performed using *scikit-learn*, *numpy*, *pandas*, *matplotlib*, *scipy*, *statsmodels*, and *seaborn* libraries in Python 3.8.0.

## Results

### Demographics and Radiological Characteristics

A total of 236 cases were enrolled in this study (**Table 2**), of which 114 cases were pathologically diagnosed as SFT/HPCs and 122 cases were pathologically diagnosed as meningiomas. There were no significant differences between the two groups in terms of gender (p = 0.770) and age (p = 0.163). Almost half the cases of both tumors occurred in the convexity, including cerebrum and cerebellum. Yet, 50 cases of meningiomas were located at the skull base compared to 32 cases of HPCs. For radiological factors, most cases showed clear tumor boundary without bone erosion. Meningiomas displayed more dural tail sign, while only 9.3% of SFT/HPCs displayed the dural tail sign. However, SFT/HPC lesions showed more invasion to venous sinus (p = 0.001) and more cystic components (p < 0.001). A heterogeneous enhancement pattern was observed in 79.6% of all SFT/HPCs and in 64.7% of all meningiomas with significant differences (p < 0.001). No significant differences in bone erosion (p = 0.39) and peritumoral edema (p = 0.361) were present.

**Table 2:** Demographic table.

	SFT/HPC (n = 114)	Meningioma (n = 122)	Total (n = 236)	*p*-value
**Age (years)**	42.72 ± 14.87	45.13 ± 11.64	43.97 ± 13.32	0.167
**Female (n, %)**	52 (45.6%)	59 (48.3%)	111 (47.0%)	0.77
**Location**				0.024
Convexity	74	58	132	
Skull base	40	50	90	
Falx	5	11	16	
Intraventricular	5	3	8	
**Boundary Clear (n, %)**	89 (78.1%)	108 (88.5%)	197 (83.5%)	0.05
**Bone Erosion (n, %)**	12 (10.5%)	8 (6.6%)	20 (8.5%)	0.39
**Dural Tail (n, %)**	10 (8.8%)	41 (33.6%)	51 (21.6%)	<0.001
**Enhancement**	n = 109	n = 119		0.011
homogeneous	21	42	63	
heterogeneous	88	77	165	
**Venous sinus invasion (n, %)**	49 (43.0%)	28 (23.0%)	77 (32.6%)	0.001
**Peritumoral edema (n, %)**	41 (36.0%)	52 (42.6%)	93 (39.4%)	0.361
**Cystic component (n, %)**	26 (22.8%)	6 (4.9%)	35 (14.8%)	<0.001

HPC, hemangiopericytoma; SFT, solitary fibrous tumor.

### Diagnostic Performance of Radiological Features and Deep Learning Model

**Figure 3:**
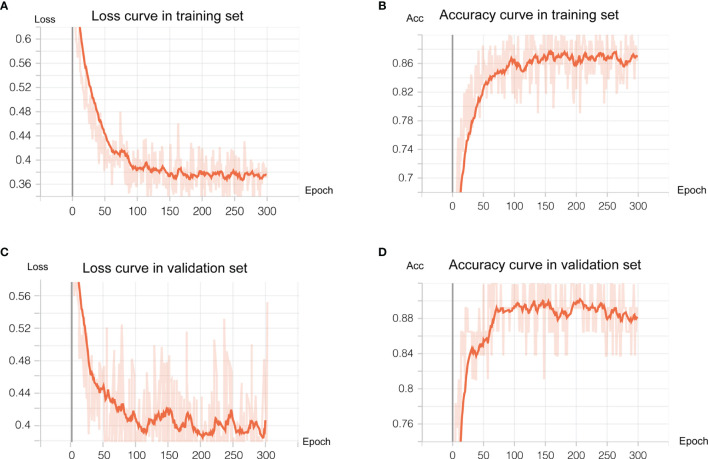
Deep learning training and validation. We trained 300 epochs and training and validation loss reached convergence at around 100 epochs. **(A, B)** showed loss curve of the transfer model in the training set and validation set, respectively. **(C, D)** showed the accuracy curve of the transfer model in the training set and validation set, respectively.

**Figure 4:**
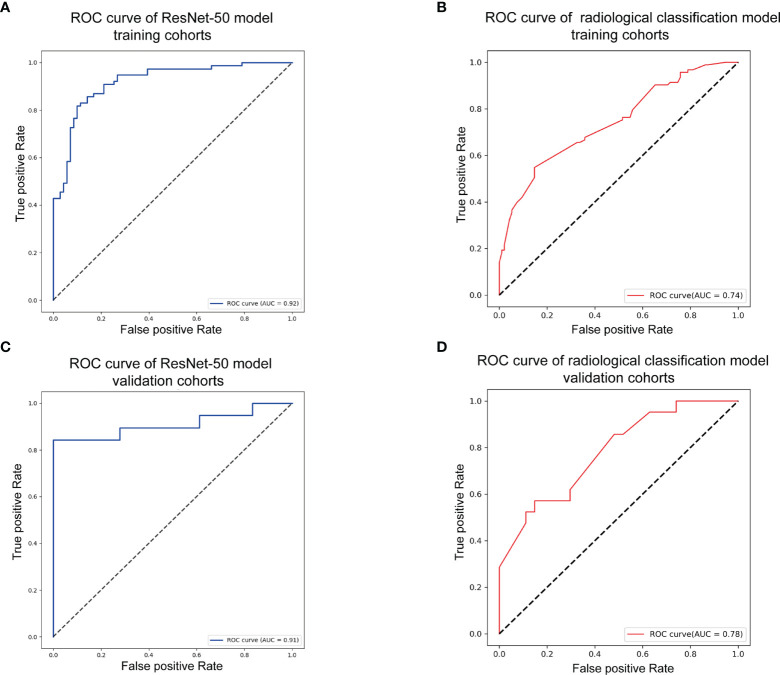
Model evaluation. **(A)** Receiver-operating characteristic (ROC) curve of the transfer model in the training set with receiver-operating characteristic curve area under the curve (AUC) of 0.92. **(B)** ROC curve of the radiological feature classification model in the training set with AUC of 0.74. **(C)** ROC curve of the transfer model in the validation set with AUC of 0.91. **(D)** ROC curve of the radiological feature classification model in the validation set with AUC of 0.78.

As shown in **Figure 3**, the transfer learning model reached a stable convergence at around 100 steps of training. After a model convergence, we got an average loss of 0.400 ± 0.040 [mean ± standard deviation (SD)] and an accuracy of 0.889 ± 0.024 in the validation set. The deep learning model reached satisfactory AUCs (**Figures 4A, C** **)** of 0.92 and 0.91 in the training and validation cohorts, respectively. In comparison, applying these radiological features (tumor boundary, bone erosion, dural sign, T1C enhancement patterns, venous sinus invasion, cystic components, and peritumoral edema) for differentiating SFT/HPC from meningiomas in our study only reached AUCs (**Figures 4B, D** **)** of 0.74 and 0.78 in the training and validation cohorts, respectively. And for the validation set, quantitative metrics were calculated and shown in **Table 3**. The model achieved higher NPV (100% for SFT/HPC and 86.00% for meningioma) and sensitivity (100% for SFT/HPC and 84.21% for meningioma) for SFT/HPC compared to meningioma. And the model achieved higher PPV (85.71% for SFT/HPC and 100% for meningioma) and specificity (84.21% for SFT/HPC and 100% for meningioma) for meningioma compared to SFT/HPC. The f1-score for both tumors was similar (0.92 for SFT/HPC and 0.91 for meningioma). The AP value for SFT/HPC was 0.92 and for meningioma was 0.86.

**Table 3:** Quantitative results for the validation set.

	PPV	NPV	Sensitivity	Specificity	F1-score	AUC	AP
**SFT/HPC**	85.71%	100%	100%	84.21%	0.92	0.91	0.92
**Meningioma**	100%	86%	84.21%	100%	0.91	0.91	0.86

HPC, hemangiopericytoma; SFT, solitary fibrous tumor; PPV, positive predictive value; NPV, negative predictive value; AUC, receiver-operating characteristic curve area under the curve; AP score, average precision score.

### Analysis of Feature Maps in Convolutional Layers

**Figure 5:**
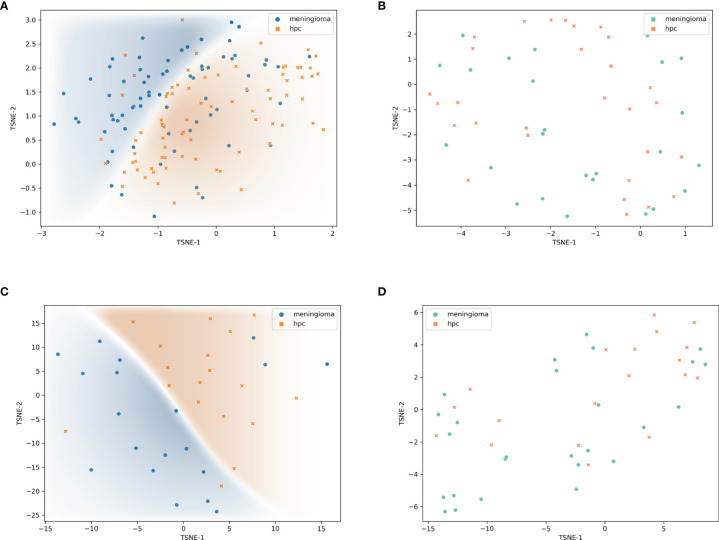
Visualization of feature maps. Features extracted by transfer model and visualized by t-distributed stochastic neighbor embedding (t-SNE) showed distinct clustering in the training set **(A)** and validation set **(C)**, respectively. Features extracted by radiological feature classification model and visualized by t-SNE showed non-distinct clustering in the training set **(B)** and validation set **(D)**, respectively.

**Figure 6:**
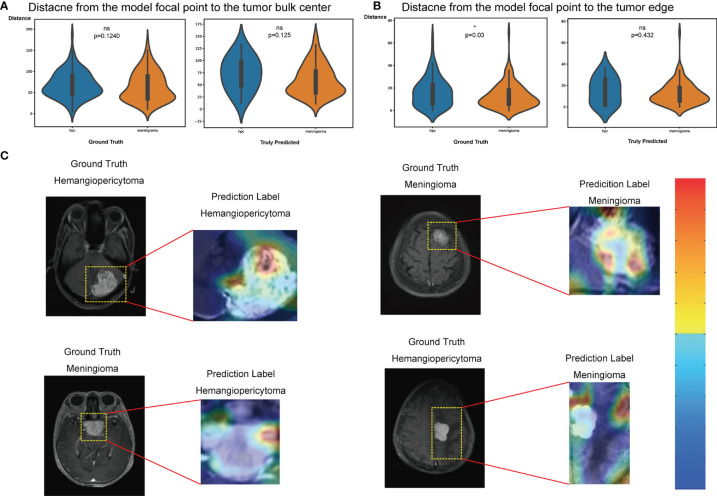
Classification activation maps. **(A)** Distance from the model’s focal point to the tumor bulk in the ground truth group (left) and truly predicted (right) group, respectively. Wilcoxon rank test, *p < 0.05. **(B)** Distance from the model’s focal point (outside the tumor) to the tumor edge in the ground truth group (left) and truly predicted (right) group, respectively. Wilcoxon rank test, *p < 0.05. **(C)** Coarse attention maps generated using Smooth Grad-CAM++ for correctly (upper row) and incorrectly (lower row) solitary fibrous tumor/hemangiopericytoma (SFT/HPC) and meningioma classification. For each pair, the postcontrast T1-weighted scan and the Smooth Grad-CAM++ attention maps (overlaid on scan) have been shown in the cropped images. In activation maps, warmer and colder colors represent high and low contribution of pixels toward a correct prediction, respectively. ns, no significance.

The feature maps we extracted represent the average pooling layer before the classification layer. Furthermore, results from the t-SNE show a distinct clustering of SFT/HPC and meningioma in the training and test cohorts (**Figures 5A, C** **)**. However, t-SNE of patients based on radiological features did not show an obvious cluster tendency of the two kinds of tumor (**Figures 5B, D** **)**. By implementing Smooth Grad-CAM++, we identified the regions within the image that mostly contributed to the prediction model (**Figure 6C**). The warm tones in the heatmap in the vicinity of the tumor show attention regions of the model. We found that for truly predicted groups, network attention overlapped with the tumor areas for SFT/HPC and meningioma. For incorrectly predicted groups, the attention regions of the model were deviated from the tumor bulks. And we calculated the distance from the attention focal point to the tumor bulk and found no significant differences between the SFT/HPC and meningioma for the ground truth group (*p* = 0.124) (**Figure 6A**, left) and the true predicted group (*p* = 0.125) (**Figure 6A**, right). Also, no significant differences in the distance were found from the focal points (when outside of the tumor) to the outer edge of the tumor for the truly predicted group (*p* = 0.432) (**Figure 6B**, right). But for the ground truth group, SFT/HPC showed a little bit higher distance from the focal points to the outer edge of the tumor (*p* = 0.03) (**Figure 6B**, left). The results suggested tumor bulks of both tumors are the attention areas for the model.

## Discussion

In our study, we established a neural network classification model to distinguish SFT/HPC from meningioma. State-of-the-art deep learning architecture based on pretrained ResNet-50 was adapted on single T1C sequence images, and it achieved a high prediction accuracy of 0.899 with AUCs of 0.92 and 0.91 in the training and validation sets. Extracting the feature maps and applying unsupervised learning also showed good performance of image feature training. Our results suggest a promising approach for automated discriminating these two types of tumors.

Intracranial SFT/HPC is a rare tumor with a diagnostic age of 35–50 years and a similar male-to-female ratio (5, 29, 30). Consistent with previous studies, our study reports that SFT/HPC and meningioma occur on similar average age and gender ratios, and we also reported a similar location distribution in convexity and skull base. Using demographic features to diagnose SFT/HPC was difficult, so physicians relied on the preoperative radiological factors to make decisions. Specifically, firstly, the “dural tail” sign, described as the thickness of the dura adjacent and traditionally considered as a specific sign (31), was significantly different in SFT/HPC and meningioma. Thus, its appearance points toward diagnosing meningioma. Secondly, intracranial SFT/HPC has a rich blood supply, leading to marked heterogeneous enhancement detected in most cases, which may be explained by pathological characteristics (13, 32, 33). Thirdly, HPC is more aggressive and tends to have more sinus invasions, cysts components, and peritumoral edema (13, 34). Yet, our study reported no significant difference in edema. On the other hand, edema and cystic properties of the tumor indicate a high malignancy and the necessity of surgical resection. We also evaluated the classification performance by using radiological features. The results of the radiological diagnostic model in our study are not good; however, these features are quite important for a preliminary clinical impression to discriminate the tumors. Lastly, other studies (14, 15; 35, 36) examined DWI and SWI characteristics of these two tumors. These studies reported higher ADC values in SFT/HPC due to its redundant vascular spaces and increased perfusion. And mean ADC values in peritumoral edema in SFT/HPC were lower. It may be speculated that the rapid growth and infiltration into adjacent normal tissue caused the edema and lower ADC values (36).

Recent advances in deep learning-assisted approaches have been explored, extracting more quantitative information from limited data. In 2019, Li etal. (36) investigated the classification of the two tumors by a radiomics approach on texture analysis. They reported an accuracy of 77.3% and 87.5% based on DWI and T1 images. However, the small sample size and lack of validation set limited the confidence of their results. Wei et al. (20) developed a clinic-radiomics diagnostic approach called Intracranial hemangiopericytoma (IHPC) and Meningioma Diagnostic Tool (HMDT). It achieved an AUC performance of 0.941 in classification of intracranial HPC and meningioma. And Dong et al. (37) also proposed similar radiomics classification methods. Compared to our model, radiomics semantic feature extraction and machine learning classification were independent and might be biased by different feature extraction and model classification approaches and researchers. And it mainly relied on feature fusion of multimodal MRI images, which limited the application in practice. Thus, our study proposed a transfer model that could combine feature extraction and prediction based on only single-modal images with a strong performance and it could accomplish an end-to-end deployment. In our training strategies, we also compared the T1 and T2 sequences with T1C, but they did not reach quite good performance. SFT/HPC and meningioma often showed enhancement in T1C, which could appear different with normal brain tissue in signal intensity. This may help the neural network to recognize the tumor patterns. T2 sequences may provide more information about peritumoral edema. However, edema surrounding tumors showed similar signals with tumors that made the boundary hard to identify. On the other hand, we lack some T2 or FLAIR scanning images because those patients could have scanned in other hospitals or only have poor image qualities, and approximative edema pattern of SFT/HPC and meningioma also increased the difficulty of classification. Thus, our single-modal model has wider implications in clinical work especially in primary hospitals, and it is easy to integrate the imaging systems.

By implementing CAMs, the attention area of the model suggested that the tumor bulk regions are quite essential for recognition. In other words, the tumor enhancement patterns play a critical role in model classification. And pseudo color reflection in our preprocessing steps also was useful for training by modifying the image contrasts. The results suggested that we should specifically focus on the enhancement regions and compare the characteristics of contrast differences. Texture features caused by abundant blood supply and necrosis in the tumor bulks made the heterogeneous enhancement patterns. Visualizing the model activation areas could assist us to pay more attention to these regions when suspecting a tumor of SFT/HPC. In addition, postcontrast images that we used are more trustworthy for clinical explanation and model understanding in clinical practice.

Our study illustrates a classification model with an improved performance. Yet, there are several limitations in our study. First of all, only patients in one particular hospital were enrolled in the study. Hence, to better extend the robustness of the model, external validation datasets need to be applied to test the model reliability. Second, DWI and SWI and even functional MRI are reliable to predict tumor types that we need to explore and excavate in future studies. Third, both kinds of tumors have distinct subgroups that require different management strategies and prognoses. Our model only reached a generalized classification. More data such as age, gender, and laboratory tests need to be combined for a more precise prediction. Lastly, considering that tumor bulk is very important for both tumor recognition, further biological and molecular characteristics would be investigated in the future.

## Conclusions

We proposed a deep learning model to classify preoperative MRI of SFT/HPC and meningioma based on single T1C modal MRI images. Our model shows high performance to distinguish the two tumor types with an average accuracy of 0.899 and AUC of 0.91 in the validation set. The tumor bulks that represent the solid texture features of both tumors are essential for model discrimination. Hence, our study paves the way toward an improved clinical diagnosis and management of these tumor diseases.

## Data Availability Statement

The data analyzed in this study are subject to the following licenses/restrictions: The data were collected from our hospital that contain the privacy of patients. Requests to access these datasets should be directed to lxjneuro@csu.edu.cn.

## Ethics Statement

The studies involving human participants were reviewed and approved by the Ethics Committee of the Xiangya Hospital, Central South University. Written informed consent to participate in this study was provided by the participants’ legal guardian/next of kin.

## Author Contributions

XL and NY designed this study, NY, ZC, QY performed the analysis, ZC wrote the manuscript, NY, NJ, SW finished the segmentation of all MRI images, ZC, NY, QY contributed in acquisition of the data used in this study. All authors read and approved the final manuscript.

## Funding

This work was supported by the National Natural Science Foundation of China (for XL, Grant No. 81770781 and No. 81472594) and the Natural Science Foundation of Hunan Province of China (for SW, Grant No. 2019JJ50978).

## Conflict of Interest

The authors declare that the research was conducted in the absence of any commercial or financial relationships that could be construed as a potential conflict of interest.

## Publisher’s Note

All claims expressed in this article are solely those of the authors and do not necessarily represent those of their affiliated organizations, or those of the publisher, the editors and the reviewers. Any product that may be evaluated in this article, or claim that may be made by its manufacturer, is not guaranteed or endorsed by the publisher.
