# Supplementary figures and images for: Deep Learning Model for Intracranial Hemangiopericytoma and Meningioma Classification
Source: Front Oncol. 2022 Mar 3;12:839567. doi: 10.3389/fonc.2022.839567 (PMC8927090; doi:10.3389/fonc.2022.839567)

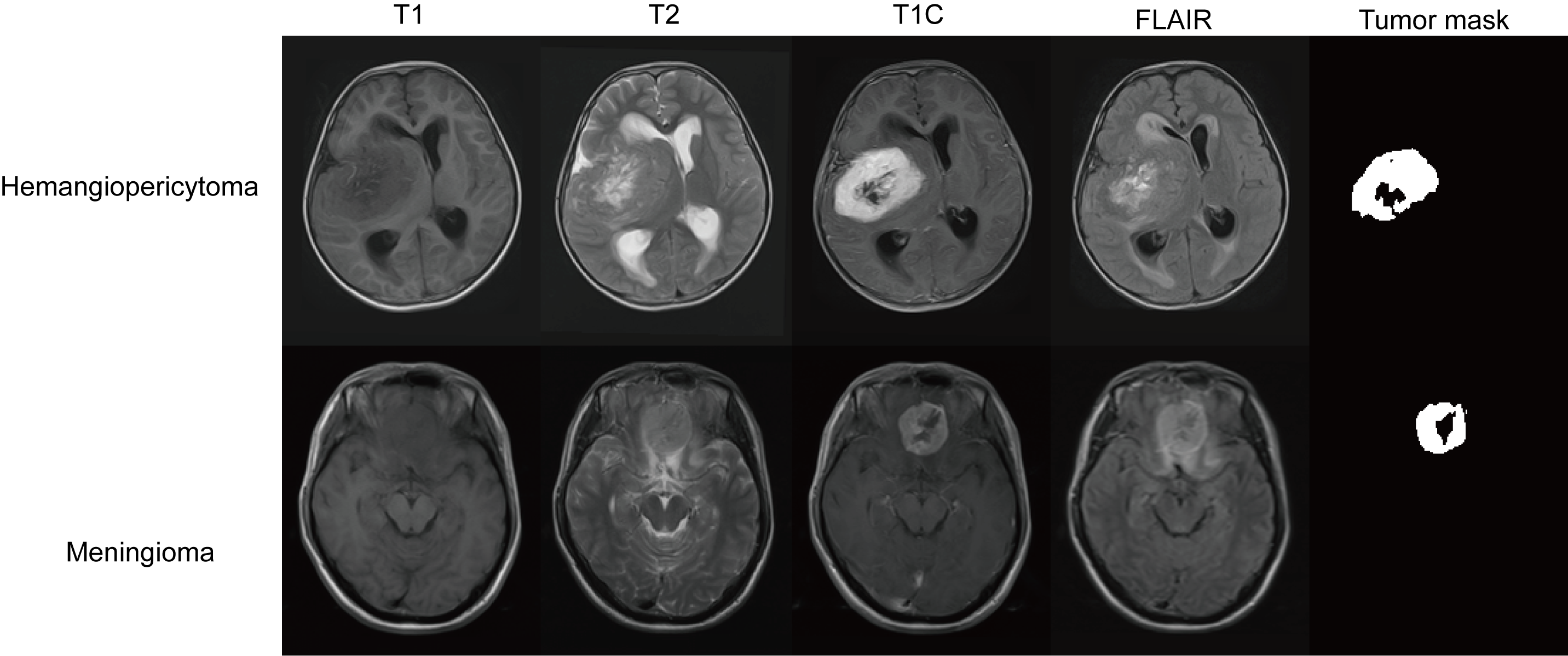

Supplement: Supplementary file 1 [file Image_1.tif]
